# Impaired encoding of rapid pitch information underlies perception and memory deficits in congenital amusia

**DOI:** 10.1038/srep18861

**Published:** 2016-01-06

**Authors:** Philippe Albouy, Marion Cousineau, Anne Caclin, Barbara Tillmann, Isabelle Peretz

**Affiliations:** 1 Lyon Neuroscience Research Center, Auditory Cognition and Psychoacoustics Team & Brain Dynamics and Cognition Team, CRNL, CNRS UMR5292, INSERM U1028, Lyon, F-69000, France; 2 Université Lyon 1, Lyon, F-69000, France; 3 International Laboratory for Brain, Music and Sound Research (BRAMS), Université de Montréal, Montreal, QC, Canada H3C 3J7; 4 Montreal Neurological Institute, McGill University, Montreal, QC Canada H3A 2B4

## Abstract

Recent theories suggest that the basis of neurodevelopmental auditory disorders such as dyslexia or specific language impairment might be a low-level sensory dysfunction. In the present study we test this hypothesis in congenital amusia, a neurodevelopmental disorder characterized by severe deficits in the processing of pitch-based material. We manipulated the temporal characteristics of auditory stimuli and investigated the influence of the time given to encode pitch information on participants’ performance in discrimination and short-term memory. Our results show that amusics’ performance in such tasks scales with the duration available to encode acoustic information. This suggests that in auditory neuro-developmental disorders, abnormalities in early steps of the auditory processing can underlie the high-level deficits (here musical disabilities). Observing that the slowing down of temporal dynamics improves amusics’ pitch abilities allows considering this approach as a potential tool for remediation in developmental auditory disorders.

Congenital amusia refers to lifelong deficits of music perception and production^1^,^2^,^3^,^4^,^5^. Unlike acquired amusia following brain damage^6^,^7^,^8^ congenital amusia occurs without brain injury, cognitive deficits, or hearing loss^3^. The most widely investigated hypothesis is that the musical deficits arise from altered pitch processing, resulting in impairments in pitch discrimination and short-term memory and sometimes associated with deficits along the musical time dimension (i.e., altered processing of rhythm or meter)^3^,^4^,^9^.

Pitch discrimination deficits have been reported using numerous behavioral paradigms^10^,^11^,^12^,^13^. Amusics exhibit difficulty detecting pitch changes in repeating tone sequences for changes smaller than two semitones, whereas controls detect changes of a quarter of a semitone^12^. Amusics show elevated pitch discrimination and pitch direction thresholds in comparison to controls, although a few amusics exhibited pitch thresholds in the range of controls’ thresholds^5^,^11^,^14^,^15^,^16^. Based on these behavioral findings, congenital amusia has been defined as a deficit in the processing of fine spectro-temporal information that cannot be explained by disorders in the auditory periphery^17^.

In addition to the perceptual impairments, short-term memory deficits for pitch have been reported. Amusics’ performance in short-term memory tasks is more affected than that of controls by increasing the duration of the retention interval between single tones^18^,^19^, by increasing the lengths of the to-be compared tone sequences^18^, and by the interference of irrelevant tones presented during the retention interval^18^,^19^. In contrast, short-term memory for verbal material has been reported unimpaired in congenital amusia when investigated with a conventional “span” memory task for numbers^20^ or with short-term memory tasks for mono-syllabic words^16^.

The relationship between pitch discrimination and short-term pitch memory deficits in amusia is under investigation. Recent studies^3^,^14^,^21^ have suggested that perceptual impairments, as revealed by discrimination tasks, can influence short-term memory deficits in congenital amusia. Indeed, amusics’ pitch memory performance improves as the discrimination between a standard and a comparison element becomes easier^14^. However, other studies indicate that short-term memory deficits persist even when the to-be-detected changes are above participants’ pitch discrimination threshold^16^,^18^,^19^,^22^,^23^.

Auditory perception and memory have been described as relying on several processing steps in which information has to be (a) extracted by the perceptual systems (extraction of auditory attributes)^24^, (b) maintained in *echoic memory*, where a pitch memory trace of the sound is established^25^,^26^, and (c) stored in auditory short-term memory for several seconds or minutes^27^. Within this framework, pitch discrimination and short-term memory might share at least the first two mechanisms. Indeed, a simple pitch discrimination paradigm requires the comparison between the *memory traces* of a previously heard stimulus and a present stimulus^28^. Similarly, in the context of a short-term memory task for tone sequences, each tone of the to-be-remembered sequence has to be kept in memory (*memory trace*). This allows for the efficient encoding of the entire sequence that can then be actively maintained in short-term memory for several seconds.

Recent neurophysiological studies in congenital amusia showed impaired encoding of tones both at the level of the brainstem and at the level of the auditory cortex. Auditory brainstem responses to complex sounds are sometimes reduced and delayed in amusic individuals as compared to controls^29^ (but see^30^). Some abnormalities have also been reported at the cortical level: when encoding the first melody of a pair in a short-term memory task, the amusic brain elicited reduced and delayed N100 m components in bilateral auditory cortices and inferior frontal gyri^22^. This was interpreted as reflecting less efficient encoding of the auditory information that might negatively impact subsequent processing steps within short-term memory, namely retention and retrieval^31^. Based on this observation, it could be hypothesized that impaired encoding of pitch information in amusia leads to the general impairments observed for pitch processing (for both pitch discrimination and memory tasks).

In previous psychoacoustic research with typical listeners, it has been shown that the capacity of discriminating and memorizing short sounds is related to the duration of the to-be-encoded material. For short sounds (<300 ms), the amount of time required to construct an appropriate memory trace of that sound can exceed the duration of the sound itself^28^. Indeed, for typical individuals, the detection of pitch changes between successive short tones is facilitated by the introduction of a silent gap (inter-tone-interval, ITI) between the tones or by increased tone durations^28^.

In the present study, we aimed to further characterize amusics’ pitch deficits by investigating to what extent discrimination and memory impairments are related to the temporal dynamic of the to-be-encoded pitch information. In Task 1, ten amusics and ten matched control participants were required to indicate whether two consecutive tones (presented without any ITI) were the same or different. We manipulated tone duration (100 ms, 350 ms) and task difficulty (pitch interval size between the tones of one or two semitones). In Task 2, the same amusic and control participants were required to compare two tone sequences separated by a 2-s delay. Over four different blocks, we manipulated tone duration (100 ms, 350 ms) and ITI (present or absent), which also resulted in changes in stimulus onset asynchrony (SOA, corresponding to the sum of tone duration and ITI). It has been demonstrated that short-term memory abilities decrease with increasing memory load for auditory and visual modalities^18^,^27^,^32^. We thus manipulated the sequence length (three or four tones) to test whether the benefits of an increased time to encode tone sequences can be observed in particular when the task difficulty increased. Note that the pitch changes for Task 2 were always larger than 3 semitones, thus above amusic participants’ pitch discrimination thresholds.

If encoding of rapid pitch information is altered in congenital amusia, we predict: a) impaired performance in amusics compared to controls when the time to encode the information is short, across both discrimination and memory tasks; b) better task performance in both amusics and controls with increased time to encode the information (duration and/or ITI, and hence SOA). Furthermore, this would also suggest that by increasing the duration of stimulus parameters (tone duration, ITI, SOA) sufficiently, amusics might be able to perform normally (at the level of controls) on pitch discrimination and memory tasks.

## Results

### Task 1: Single tone comparison

**Figure 1:**
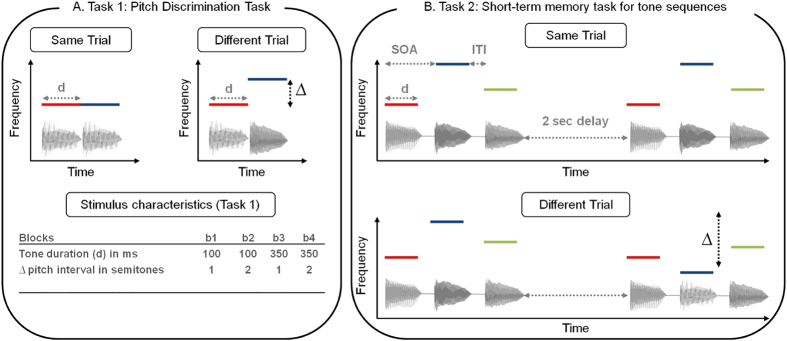
(**A**) Stimulus characteristics and examples of same and different trials for the single tone comparison task. Tones were presented with no ITI. (**B**) Examples of same and different trials for the short-term memory task for the sequence length of 3 tones. For “same” trials, S1 was repeated as the second melody of the pair (S2) after a 2000 ms silent delay. For “different” trials, one tone in S2 was altered to change the melodic contour. Tones are represented as waveforms and their fundamental frequencies are illustrated with colored lines.

In Task 1, participants had to determine whether two tones (played with a piano timbre) presented without an ITI were the same or different (Fig. 1A). The task was divided into four blocks between which tone duration (d = 150 or 350 ms) and pitch interval sizes (∆ = one or two semitones) were manipulated.

**Figure 2:**
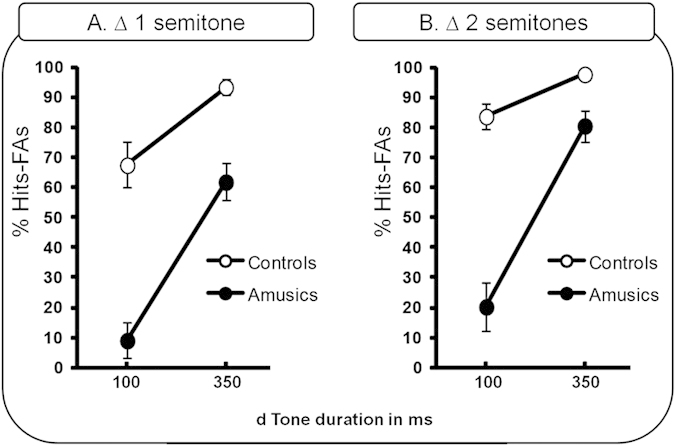
Amusics’ and controls’ performance for the Single Tone Comparison Task (Task 1) presented as a function of task difficulty (pitch interval size: (A). one semitone, (**B**) two semitones) and stimulus characteristics (tone duration: 100 ms short, 350 ms long). Black circles, amusics; white circles, controls. Error bars indicate the standard errors of the means, SEM.

Percentages of Hits-FAs (Fig. 2) were analyzed with a 2 × 2 × 2 ANOVA with group (amusics, controls) as the between-participants factor and tone duration (100 ms or 350 ms) and pitch interval size (∆ = one semitone or two semitones) as within-participant factors.

The main effect of group was significant [*F*(1,18) = 44.15 ; *P* < 0.0001; *MSE* = 824.2; *η*^*2*^_*p*_ = 0.71], with poorer performance on average in amusics (mean = 42.93%; SD = 35.62) than in controls (mean = 85.63%; SD = 18.21). The main effect of tone duration was also significant [*F*(1,18) = 175.22 ; *P* < 0.0001; *MSE* = 167.3; *η*^*2*^_*p*_ = 0.90] with better mean performance for the long tone duration (d = 350 ms; mean = 82.44%; SD = 19.31) than for the short tone duration (d = 100 ms; mean = 45.16%; SD = 37.54). There was a significant main effect of pitch interval size [*F*(1,18) = 20.03 ; *P* < 0.0001; *MSE* = 159.9; *η*^*2*^_*p*_ = 0.52], with performance for the larger pitch interval size (∆ = 2 semitones; mean = 70.63%; SD = 34.18) better than performance for the smaller pitch interval size (∆ = 1 semitone; mean = 57.97%; SD = 35.82).

A significant interaction was found between tone duration and group [*F*(1,18) = 39.96; *P* < 0.0001; *MSE* = 167.3; *η*^*2*^_*p*_ = 0.68], and this was modulated by pitch interval size [*F*(1,18) = 4.37; *P* = 0.05; *MSE* = 107.2; *η*^*2*^_*p*_ = 0.19]. While amusics’ performance was significantly decreased in comparison to controls for the most difficult block (d = 100 ms, ∆ = one semitone, *P* < 0.0001), this was not the case for the easiest block (d = 350 ms, ∆ = two semitones, *P* = 0.08, see Fig. 2). Moreover, while amusics showed better performance for the long tone duration in comparison to the short tone duration for both pitch interval sizes (all *P-values* < 0. 0001), controls showed this pattern only for the smaller pitch interval size (∆ = one semitone, *p* = 0.05) and not for the larger interval size (∆ = two semitones, *p* = 0.35)). This latter effect can be related to a ceiling performance for the larger pitch interval size, for which controls’ performance was not significantly different from a perfect score (100%, all *P-values* > 0.06).

To assess the potential differential benefit of tone duration and pitch interval size in both groups and between groups, we performed the following subtractions on the %Hits-FAs data:

1. block with [*d-100 ms*, ∆*-2semitones*] minus block with [*d-100 ms*, ∆*-1semitone*] to investigate the benefit of pitch interval size in short tones;
2. block with [*d-350 ms*, ∆*-1semitone*] minus block with [*d-100 ms*, ∆*-1semitone*] to investigate the benefit of tone duration for small pitch changes.

The resulting data were analyzed with a 2 × 2 ANOVA with group as a between-participant factor and type of benefit (pitch interval size, tone duration) as within-participant factors.

The main effect of group was not significant [*F*(1,18) = 1.82; *P* = 0.19; *MSE* = 655.11; *η*^*2*^_*p*_ = 0.09]. The main effect of type of benefit was significant [*F*(1,18) = 45.93; *P* < 0.0001; *MSE* = 142.96; *η*^*2*^_*p*_ = 0.71], and was further modulated by group [*F*(1,18) = 17.76; *P* < 0.0001; *MSE* = 142.96; *η*^*2*^_*p*_ = 0.49]. While amusics showed a stronger benefit of increased tone duration than did controls (*P* = 0.005), the benefit of increased pitch interval size did not differ between the two groups (*P* = 0.58). In addition, the benefit of increasing tone duration was significantly greater than the benefit of increasing pitch interval size in amusics (*P* < 0.0001), while this was not the case in controls (*p* = 0.08). Amusics’ performance thus benefited more from increasing stimulus duration than from increasing pitch change. This suggests that the temporal dynamic of pitch information has a more important impact than the spectral information on amusics’ pitch discrimination abilities.

### Task 2: Short-term memory tasks for tone sequences

**Figure 3:**
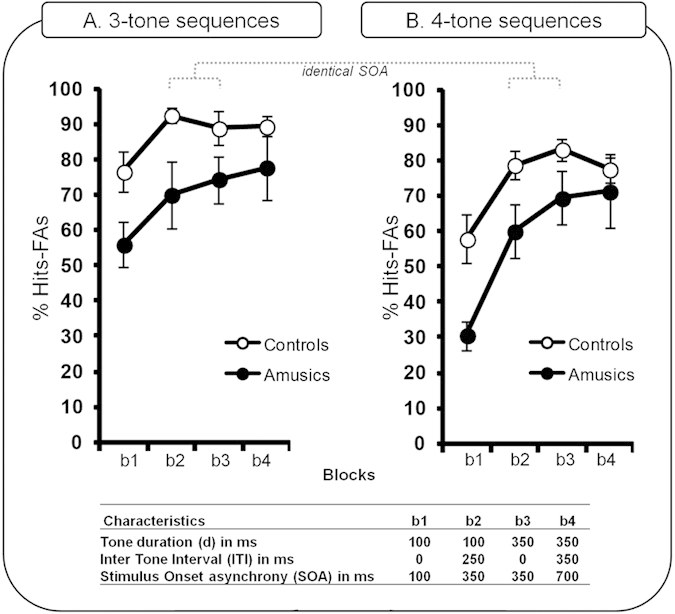
Amusics’ and controls’ percentage of Hits-False Alarms in the short-term memory task (Task 2), presented as a function of task difficulty (sequence length: (A). three-Tone Sequence; (**B**) four-Tone Sequence) and blocks varying in tone duration, inter-tone-interval, and stimulus onset asynchrony. Black circles, amusics; white circles, controls. Error bars indicate the standard errors of the means, SEM.

In Task 2, participants performed a melodic short-term memory task for which they had to compare two tone sequences (S1 and S2; played with a piano timbre) separated by a silent retention period of 2000 ms (Fig. 1B). To manipulate task difficulty, sequences were composed of either of three or four tones. For each sequence length, there were four blocks differing in tone duration and/or ITI. The duration of the tones was either 100 ms (b1 and b2) or 350 ms (b3 and b4), and they were presented either without an ITI (b1 and b3) or with an ITI (b2 and b4), resulting in a range of different SOAs (see Fig. 3). Note that for blocks b2 and b3, the SOA between tones was equal, allowing us to disentangle the contribution of tone duration and SOA in short-term memory performance.

%Hits-FAs were computed as described above. Data were analyzed with a 2 × 2 × 2 × 2 ANOVA with group (amusics, controls) as the between-participants factor and sequence length (three-tones, four-tones), tone duration (100 ms, 350 ms), and ITI (present, absent) as within-participant factors. The data are summarized in Fig. 3.

The main effect of group was significant [*F*(1,18) = 6.02; *P* = 0.02; *MSE* = 1838.0; *η*^*2*^_*p*_ = 0.25], with better performance in controls (mean = 80.54%; SD = 16.73) than in amusics (mean = 63.67%; SD = 27.59). As expected, the main effect of sequence length was significant [*F*(1,18) = 43.30; *P* < 0.0001; *MSE* = 141.1 *η*^*2*^_*p*_ = 0.70], with participants showing better performance for three-tone sequences (mean = 78.17%; SD = 22.19) than for four-tone sequences (mean = 66.05%; SD = 24.86). There was a significant main effect of ITI [*F*(1,18) = 7.34, *p* = 0.01, *MSE* = 577.1; *η*^*2*^_*p*_ = 0.28], with all participants showing better performance for trials with an ITI (mean = 77.37; SD = 21.84) than for trials without an ITI (mean = 67.08; SD = 24.71). The main effect of tone duration was also significant [*F*(1,18) = 37.59, *P* < 0.0001, *MSE* = 192.8; *η*^*2*^_*p*_ = 0.67] and further modulated by group [*F*(1,18) = 5.49, *P* = 0.03, *MSE* = 192.8; *η*^*2*^_*p*_ = 0.23]. Post hoc tests revealed that while amusic and control groups both showed the benefit of longer tone duration over short tone duration (amusics: *P* < 0.0001; controls: *P* = 0.01; see Fig. 3), amusics’ performance was decreased (in comparison to controls) for the short tone duration (100 ms; *P* = 0.005, Fig. 3, b1 and b2), but not for the long tone duration (350 ms; *P* = 0.17, Fig. 3, b3 and b4).

Other significant interactions did not involve the group factor, notably the sequence length by tone duration interaction [*F*(1,18) = 5.26, *P* = 0.034, *MSE* = 194.9; *η*^*2*^_*p*_ = 0.22], as well as the tone duration by ITI interaction [*F*(1,18) = 52.88, *P* < 0.0001, *MSE* = 79.8; *η*^*2*^_*p*_ = 0.74]. In addition, the three-way tone duration by sequence length by ITI interaction was significant [*F*(1,18) = 6.94, *P* = 0.01, *MSE* = 61.0; *η*^*2*^_*p*_ = 0.27]. To analyze this 3-way interaction, post hoc tests were performed and revealed that for the easier task (three-tone sequence length), both groups exhibited similar performance for short tones with an ITI and for long tones with or without an ITI (all *P-values* = 0.44). However, for the more difficult task (four-tone sequence length), performance for short tones with an ITI was reduced in comparison to performance for the long tone duration with or without an ITI (all *P-values* < 0.01). Additionally, this analysis revealed that for both sequence lengths (three-tones, four-tones), amusics and controls’ performance was increased with the presence of an ITI only for the short tone duration (three-tones, *P* < 0.0001; four-tones, *P* < 0.0001) and not for the long tone duration (three-tones, *P* = 0.44; four-tones, *P* = 0.46).

### Correlations

Two sets of correlations were done. First we performed the correlation between data of Task 1 and 2 to investigate the link between pitch discrimination and pitch memory. Correlations involving data from Tasks 1 and 2 were computed with the average of %Hits-FAs over all conditions (of each task, respectively). We then correlated data of Tasks 1 and 2 with behavioral data from previous testing sessions (Montreal Battery of Evaluation of Amusia^33^ and Pitch Change Detection task (PCD, see Table 1) from Hyde and Peretz^12^ (see also^1^,^34^,^35^,^36^)). This analysis aimed to investigate whether participants’ scores on diagnostic tests (that require both discrimination (PCD) and memory (MBEA)) can predict their performance on Tasks 1 and 2.

The results from Tasks 1 and 2 were positively correlated across all participants (*r*(18) = 0.77, *P* < 0.001), as well as in control participants (*r*(8) = 0.79, *P* = 0.007), and amusic participants (*r*(8) = 0.74, *P* = 0.01), considered separately (see Supplementary information Figure 1A).

Average scores on the melodic subtests of the MBEA were positively correlated with Task 1 (see Supplementary information Figure 1B) and 2 (see Supplementary information Figure 1C) results across all participants (Task1: *r*(18) = 0.85; *P* < 0.0001; Task 2: *r*(18) = 0.66; *P* = 0.001) and in amusics (Task1: *r*(8) = 0.79, *P* = 0.06; Task 2: *r*(8) = 0.88; *P* = 0.001), but not in controls *P* > 0.05).

Data of Task 1 were positively correlated with data of the PCD over all participants and in amusics for the 1/4 (see Supplementary information Figure 1D) and 1/2 semitone (see Supplementary information Figure 1E). pitch interval sizes (all participants: PCD 1/4 : *r*(18) = 0.90, *P* < 0.0001; PCD 1/2: *r*(18) = 0.71; *P* < 0.0001; amusics: PCD 1/4 : *r*(8) = 0.87, *P* = 0.001; PCD 1/2: *r*(8) = 0.65; *P* = 0.001).

Finally, data of Task 2 were positively correlated with data of the PCD over all participants (*r*(18) = 0.65, *P* = 0.002) and in amusics for the 1/4 semitone pitch interval size (*r*(8) = 0.69, *P* = 0.027) (see Supplementary information Figure 1F).

## Discussion

The present study investigated whether amusics’ deficits in pitch discrimination and short-term memory are related to impaired encoding of rapid auditory information. For both tasks, amusics exhibited impaired performance compared to controls when the time to encode pitch information was short. Amusics and controls showed pronounced improvements in terms of accuracy with increasing tone duration and/or ITI. These benefits were observed independently of the task difficulty (pitch interval size for Task 1; sequence length for Task 2). Most interestingly, when enough time was given to encode the pitch information (350 ms and more), amusics were able to reach normal performance in both tasks.

Task 1 investigated whether tone duration (equal to SOA in this task, as there was no ITI) could affect amusics’ pitch discrimination abilities for two different task difficulties (i.e., different pitch interval sizes). Amusic participants showed decreased performance in comparison to controls, but this impairment was dependent on tone duration. While amusics exhibited decreased performance for the short tone duration (d = 100 ms), their performance did not differ from that of controls for the long tone duration (d = 350 ms). It is relevant to note, however, that this latter effect might possibly be an artifact due to a ceiling effect in controls. Nevertheless, the crucial role of tone duration in amusics’ pitch discrimination abilities materialized in the comparison of the benefit of tone duration and of pitch interval size, respectively. We observed that the increase in tone duration had a stronger benefit on amusics’ performance than the increase in pitch interval size, whereas for the chosen parameters, the two benefits were not significantly different in controls.

The benefit of increasing tone duration on participants’ performance is in line with numerous psychoacoustic studies for typical listeners^37^,^38^,^39^,^40^,^41^,^42^,^43^ showing that auditory discrimination abilities benefit from increasing SOA (or here, tone duration). This effect can be interpreted in terms of reduced ‘backward masking effect’^28^,^38^,^40^,^44^ when the time to encode the information is long enough. When normal listeners are given the several hundreds of milliseconds needed to construct a proper memory trace of the pitch of a newly heard sound, the representation of this sound is optimal. In contrast, if a second sound is presented too soon after the first one, the perceptual analysis of the first sound would be prematurely stopped (‘backward masking effect’).

In agreement with these principles described for normal listeners, Task 1 showed that amusics’ pitch processing benefitted from long tone durations (as recently suggested by^17^), and by extension, a slower rate of presentation of tones. The findings that amusics exhibit decreased performance compared to controls for the short tone duration, but not for the long tone duration, and that the benefit of increasing tone duration is stronger for amusics than for controls suggests that the time constraints for pitch encoding might differ between the two groups. Amusics may need more time than controls to properly encode the sounds (construct a proper *memory trace*). The longer tones would allow for such reliable representations of pitch to be formed, and would in turn lead to increased discrimination capacity.

The importance of time to encode pitch information in congenital amusia is consistent with previous studies, notably those investigating pitch thresholds. Table 2 lists these prior studies as a function of SOA. When performing the correlation between 19 threshold values available in these studies (we considered only values that were available in the main text or in tables of the articles, see Table 2) and SOA, a clear pattern emerges despite the fact that these studies used diverse materials and different tasks. Amusics’ pitch thresholds are negatively correlated with the duration of SOA (*r*(17) = −0.48, *p* = 0.03) (Note that this effect is not significant in controls (*r*(17) = −0.34, *p* = 0.15)). While amusics exhibit increased (worse) pitch thresholds in comparison to controls for all SOAs (except for unresolved harmonics- see^17^), their threshold values are getting better (lower) when the SOA between the to–be-compared tones increases (pitch thresholds varying from 4.72 semitones with short SOA (150 ms) to 0.28 semitones for long SOA (1200 ms). This implies a beneficial impact of the temporal presentation rate of tones on amusics’ pitch discrimination abilities. Data of Task 1 and of the studies listed Table 2 thus suggest that amusics exhibit altered encoding of rapid auditory information that impairs their performance in tasks requiring pitch discrimination (as well as pitch change detection or pitch direction judgments). Moreover, they suggest that increasing the time to encode pitch information facilitates simple pitch processing in congenital amusia, with amusics able to reach performance levels that are comparable to those of controls when more time to encode the sounds is available (see Table 2 and Supplementary information Table A).

In addition to evaluating the impact of stimulus duration on amusics’ performance in a single pitch discrimination task, the present study investigated whether this parameter can affect amusics’ short-term memory abilities for melodies. Task 2 investigated whether participants’ short-term memory performance could vary as a function of tone duration (100 ms, 350 ms), ITI (present or absent), and SOA for two different task difficulties (3-tone and 4-tone sequences).

For both sequence lengths, tone duration and ITI had an impact on participants’ performance. Amusic and control groups showed better accuracy for 1) the long tone duration than for the short tone duration, and 2) tone sequences with an ITI (see Fig. 3 and Supplementary information Table B) as compared to sequences without any ITI. Furthermore, our data revealed that tone duration might have a more critical impact than SOA on participants’ performance. While accuracy was similar for blocks with the same SOA (*[short tones with ITI]* and *[long tones without ITI]*) for the easier condition (three-tone sequences), this was no longer the case for the difficult condition (four-tone sequences). For this latter condition, participants’ performance for the block *[long tone duration without ITI]* was better than for the block *[short tone duration with ITI]*. This suggests that when task difficulty increases, long tone duration is more useful for properly encoding the auditory information than is the addition of a silent gap after a short tone. More interestingly, this critical role of tone duration interacted with participant groups. While amusics showed strongly impaired performance in comparison to controls for the short tone duration, they were performing as well as controls for the long tone duration.

Based on the findings in both tasks and on the positive correlations observed between data from Tasks 1 and 2 (and with data from the pre-tests), we argue that congenital amusics’ deficits in both single pitch discrimination and short-term memory tasks are underlined by impaired encoding of rapid auditory information. These results are remarkably similar to those reported in other developmental disorders, such as dyslexia, specific language impairment (SLI)^45^,^46^,^47^ and language learning impairments (LLI)^48^. Indeed, deficits in rapid auditory processing (RAP theory) have been described in these disorders, based on their difficulty in processing brief, rapidly changing acoustic information^45^,^46^,^47^,^48^,^49^. The earliest indication of this phenomenon was the finding that the performance of language impaired and dyslexic children is inferior to that of control participants in tone-sequence tasks when the SOAs are below 400 ms, but performance was normal at longer SOA^46^,^47^. Additionally, research investigating children with specific language impairment^50^,^51^ has demonstrated that the children’s ability to process rapidly arriving (within a time window of ~40 ms) auditory information is impaired as compared to that of control children. More recently, longitudinal and cross-sectional studies have shown that basic temporal processing discrimination in infants predicts later language outcomes^52^,^53^. Based on these findings, it has been proposed that deficits in the ability to perceive rapidly changing acoustic differences are either a cause (see^54^ for review) or a consequence^55^ of language impairments, affecting language comprehension and reading ability. Observing a similar pattern of results in congenital amusics is of interest because it suggests that while these deficits are not observable for the same material (music for amusia, speech for specific language impairment and dyslexia), developmental difficulties in each of these disorders are related to altered processing of auditory information that arrive rapidly and sequentially. Further work is thus necessary to understand the potential relationships between amusia, dyslexia and specific language impairment.

Finally, given the similar performance between amusics and controls when the time to encode pitch sequences is long (350 ms and more), it could be argued that slowing down the presentation of the pitch information might improve amusics’ musical abilities. However, this hypothesis can be challenged, especially when considering that the long tone duration (350 ms) used in the present study is similar to: 1) the profile of tone durations used in Western tonal music, 280 ± 291 ms^56^ and 2) the average duration of tones constituting the non-polyphonic melodies in the MBEA (mean = 379.8 ms ± 0.19 ms), for which amusics exhibit strong deficits^33^.

When considering previous studies investigating short-term memory processing for melodies in congenital amusia (see Table 3), it can be hypothesized that other parameters, such as sequence length and/or the duration of the silent retention delay, might further influence amusics’ short-term memory performance. In the present study, the tone sequences were composed of only 3 or 4 tones, thus constituting rather simple material in terms of melodic information and memory load. In previous studies, as listed in Table 3, the sequences were longer (5 tones in Gosselin^16^,^18^,^23^; 6 tones in^22^; 7 to 21 in the MBEA Peretz^33^), and therefore more complex. It may be that slowing down the pitch information is sufficient to help the encoding of short sequences, but would not be sufficient for longer sequences. In other words, long tone duration might not fully restore a ‘normal pitch memory trace’, but could lead to a trace that is nevertheless sufficient for encoding less complex material. Further work is needed to understand the precise role of memory load and length of retention period on amusics’ pitch processing abilities for more complex musical materials.

The present study showed that amusics’ deficits in pitch discrimination and memory are related to an impaired encoding of rapid auditory information. More research is now necessary to understand the potential relationships between congenital amusia and other disorders that exhibit similar patterns of temporal deficits (eg., developmental language disorders) and to ascertain whether similar difficulties in processing rapidly presented auditory signals can be generalized to other types of material (such as speech) in congenital amusia.

## Materials and Methods

### Participants

Ten amusic participants and ten non-musician control participants matched for age, handedness, educational background (years of education), and musical training (years of musical instruction: teaching/practice) participated in the study. The amusic group (age range: 62 to 72 years) and the control group (age range: 61 to 75 years) were right-handed francophone participants from Montreal and surrounding areas. Participants reported no history of neurological or psychiatric disease, and had audiometric thresholds below 30 dB HL for frequencies below or at 4 kHz. Data from the pre-tests are presented in Table 1 and Supplementary information Table A. Participants gave their written informed consent and were paid for their participation. The research was carried out in accordance with approved guidelines of the Comité d'éthique de la recherche en arts et en sciences (CERAS) of the Université de Montréal. Ethical approval was obtained from the CERAS committee of the Université de Montréal.

### Equipment

The experiments took place in a sound-attenuated booth, and auditory stimuli were presented via SENNHEISER HD 280 pro headphones at 65 dB SPL. Presentation software (Neurobehavioral Systems, Berkeley, CA, USA) was used to control the stimulus presentation and record participants’ responses in the form of mouse button presses.

### Task 1: Single Tone Comparison

There were 64 trials (32 same, 32 different) in each block. For different trials, the second tone was equiprobably upper or lower in pitch than the first tone by ∆. The choice of these pitch interval sizes was based on results of our participants on the PCD task (d = 100 ms, ITI = 250 ms, SOA = 350 ms) showing that they reached normal pitch detection performance for pitch interval sizes of one semitone and larger (Table 1). Note that this pattern of results differs from that described in^4^ and^1^, where amusics showed decreased performance in comparison to controls for the one-semitone interval. The amusic group participating in the present study thus exhibit better pitch change detection abilities than the group from these previous studies (see Supplementary information Table A).

Tone pairs were created using eight piano tones differing in pitch height (Cubase software, Steinberg), but all belonging to the key of C Major (C3, D3, E3, F3, G3, A3, B3, C4, frequency range from 130.81 Hz to 261.63 Hz). This material was chosen to allow for comparisons between Task 1 and Task 2 (as Task 2 was a tonal melodic task, see below).

### Task 2: Short-term memory task with tone sequences

For each sequence length (three-tone sequences, four tone sequences) 128 different diatonic melodies (presented as the first sequence, S1) were created using the piano tones of Task 1. For each block, there were 32 trials (S1, silence, S2), with 16 *same* and 16 *different* trials. Participants were asked to indicate whether S1 and S2 were the same or different. For *different* trials, one tone in the S2 melody was different from the S1 melody and created a contour-violation in the tone sequence. The change on *different* trials occurred in position two or three of the sequences, regardless of sequence length, and the change position was equally distributed across trials.

For *different* trials, the pitch interval sizes (see ∆ in Fig. 2B.) were larger than 3 semitones (thus, above amusic participants’ discrimination thresholds) and controlled in such a way that no significant differences of interval sizes were observed between blocks and sequence lengths (all *P-values* >0.10; mean pitch interval size across blocks = 6.7 semitones; SD = 1.1 semitones).

### Procedure

Participants performed Task 1 first, followed by Task 2, on the same day of testing, and the entire session lasted approximately one hour. For each task, the order of presentation of the blocks was counterbalanced across participants (latin square), and blocks were separated by breaks of 2-3 minutes. Participants were informed of the stimulus characteristics before a given block: tone duration (short, long) and pitch interval size (small, large) for Task 1; sequence length (3-tone, 4-tone), tone duration (short, long), and ITI (present, absent) for Task 2. They were asked to respond by mouse button presses with their right hand after the end of the auditory stimulation. There was no time limit to respond, and participants pressed the middle mouse button to launch the next trial. Before each block, participants performed four practice trials with error feedback, but no feedback was given during the experiment. Within each block, the trials were presented in a pseudo-randomized order: the same trial type (i.e. same, different) could not be repeated more than three times in a row.

### Statistical analyses

Statistical analyses were conducted with Statistica (StatSoft, Inc, Tulsa, OK, USA). Performance in both tasks was evaluated as percentages of Hits (correct responses for *different* trials/ number of *different* trials) minus percentages of False Alarms (FAs, incorrect responses for *same* trials/number of *same* trials). Percentages of Hits-FAs were analyzed with a repeated measure ANOVA. To analyze significant interaction, post hoc tests were performed using Fischer LSD. For all the measures of interest we tested if the samples (controls and amusics) were derived from a population normally distributed using Kolmogorov-Smirnov tests. These analyses revealed that all data were normally distributed (*P* > 0.20). Furthermore, we computed correlations over all participants and for the amusic and control group, separately.

## Additional Information

**How to cite this article**: Albouy, P. *et al.* Impaired encoding of rapid pitch information underlies perception and memory deficits in congenital amusia. *Sci. Rep.* **6**, 18861; doi: 10.1038/srep18861 (2016).

## Supplementary Material

Supplementary Information

## Figures and Tables

**Table 1:** Demographic characteristics and behavioral pretest data for amusic and control participants. Means (with standard deviations in parentheses) are reported for each group and compared with independent two-sided t-tests.

Characteristics	Amusics (n = 10)	Controls (n = 10)	t-test
*Age in years*	67.10 (3.5)	65.10 (4.4)	*t*(*18*) = *1.12, P* = 0.*27*
*Gender*	5 female, 5 male	7 female, 3 male	*N/A*
*Education in years*	16.5 (2.5)	15 (3.8)	*t*(*18*) = *1.04, P* = 0.*30*
*Musical education in years*	1.4 (0.9)	1.60 (1.4)	*t*(*18*) = *0.36, P* = 0.*71*
MBEA^1^ (cut-off based on^33^)			
*Total score* (*cut-off 23.4*)	19.7 (2.6)	26.75 (1.1)	*t*(*18*) = *4.79, P* < 0.*0001*
*Melodic sub-tests score* (*cut off 21.6*)	18.2 (2.6)	27.4 (1.9)	*t*(*18*) = *8.93, P* < 0.*0001*
Pitch Change Detection^2^			
*1/4 semitone*	31.9 (21.1)	91.3 (11.1)	*t*(*18*) = *7.87, P* < 0.*0001*
*1/2 semitone*	70.5 (22.7)	94.2 (9.5)	*t*(*18*) = *3.05, P* = 0.*006*
*1 semitone*	89.3 (14.3)	94.4 (9.8)	*t*(*18*) = *0.98, P* = 0.*34*
*2 semitones*	95.5 (3.6)	94.8 (10.7)	*t*(*18*) = *0.19, P* = 0.*85*
*3 semitones*	95.7 (4.7)	94.8 (9.7)	*t*(*18*) = *0.26, P* = 0.*79*

^1^MBEA = Montreal Battery of Evaluation of Amusia, Results for the MBEA are expressed as the number of correct responses (averaged over the six tests of the battery, and the six melodic tests of the MBEA (Scale, Contour, Interval; maximum score = 30).

^2^Pitch Change Detection (PCD) scores were calculated in terms of percentage of Hits (correct response in different trials) minus percentage of false alarms (FA) in each group of participants. In the PCD task, participants are required to detect a pitch change of 1/4 semitone up to 3 semitones within the context of a five-tone sequence (isochronous, played at a pitch level of C6 (1047 Hz), tone duration = 100 ms; ITI = 250 ms, SOA = 350 ms; see^12^).

**Table 2:** Stimulus characteristics in terms of SOA, tone duration (d), and ITI in ms from previous studies in congenital amusia investigating pitch discrimination and direction thresholds.

SOA ms	d ms	ITI ms	Amusics’ threshold	Controls’ threshold	Impaired	Task	Material	n	Study
150	150	0	1.21 ST	0.34 ST	X	DLF RH	Band-passed click trains	10	Cousineau *et al.* 2015^17^
150	150	0	4.72 ST	3.55 ST		DLF UH	Band-passed click trains	10	Cousineau *et al.* 2015^17^
350	350	0	0.39 ST	0.17 ST	X	DLF RH	Band-passed click trains	10	Cousineau *et al.* 2015^17^
350	350	0	2.68 ST	1.88 ST		DLF UH	Band-passed click trains	10	Cousineau *et al.* 2015^17^
350	250	100	>1ST	<1ST	X	PDT	Discrete pure tones	10	Foxton *et al.* 2004^11^
350	250	100	<1ST	<1ST	X	PDT	Gliding pure tones	10	Foxton *et al.* 2004^11^
350	100	250	1.32 ST	0.57 ST	X	PDT	Pure tones	10	Tillmann *et al.* 2009^16^
350	100	250	1.07 ST	0.31 ST	X	PDT	Pure tones	9	Albouy *et al.* 2013^22^
350	100	250	1.32 ST	0.25 ST	X	PDT	Pure tones	11	Albouy *et al.* 2013^23^
350	100	250	1.12 ST	0.24 ST	X	PCD	Pure tones	16	Albouy *et al.* 2015^34^
500	200	300	1.18 ST	0.20 ST	X	PDT	Pure tones	14	Jiang *et al.* 2013^14^
750	250	500	0.60 ST	0.12 ST	X	DLF	Pure tones	35	Jones *et al.* 2009^57^
1200	600	600	0.28 ST	0.15 ST	X	PCD	Pure tones	16	Liu *et al.* 2010^15^
1200	600	600	0.29 ST	0.14 ST	X	PCD	Pure tones	14	Omigie *et al.* 2013^58^
1200	600	600	0.28 ST	0.15 ST	X	PCD	Pure tones	16	Williamson *et al.* 2012^59^
Pitch Direction Threshold									
350	250	glide 100	>1ST	<1ST	X	PDIT	Gliding pure tones	10	Foxton *et al.* 2004^11^
500	250	250	1.90 ST	0.19 ST	X	PDIT	Gliding complex tones	16	Liu *et al.* 2012^60^
									Liu *et al.* 2015^61^
500	250	250	4.44 ST	0.30 ST	X	PDIT	Discrete complex tones		Liu *et al.* 2012^60^
									Liu *et al.* 2015^61^
500	200	300	3.90 ST	1.01 ST	X	PDIT	Pure tones	14	Jiang *et al.* 2013^14^
1200	600	600	0.86 ST	0.20 ST	X	PDIT	Pure tones	16	Liu *et al.* 2010^15^
1200	600	600	1.68 ST	1.18 ST	X	PCD	Pure tones	16	Omigie *et al.* 2013^58^
1200	600	600	0.28 ST	0.15 ST	X	PCD	Pure tones	16	Williamson *et al.* 2012^59^

*DLF, Difference Limen Frequency; RH, Resolved Harmonics; UH, Unresolved Harmonics; PDT, Pitch Discrimination Threshold; PCD, Pitch Change Detection; PDIT, Pitch Direction Threshold. ST, Semitone. Amusics are considered impaired compared to controls when the difference between the groups was significant.*

**Table 3:** Stimulus characteristics from previous studies in congenital amusia investigating short-term memory for tone sequences in terms of SOA, tone duration (d), ITI, retention delay between melodies, Sequence Length, and average Pitch interval Size (PIS) of the change in melodies to be compared.

SOA ms	d ms	ITI ms	Delay s	Sequence Length	PIS	Amusics’ performance	Controls’ performance	Accuracy	Impaired	Unimpaired	Task	Material	n	Study
250	250	0	1.1	4	1.1 ST	75.06	95.37	% correct	x		Contour	pure tones	10	Foxton *et al.* 2004^11^
						74.87	92.06		x		Interval	pure tones		Foxton *et al.* 2004^11^
						59.12	80.43		x		Contour Transposed	pure tones		Foxton *et al.* 2004^11^
250	250	0	2	6	3ST	41.49	84.78	% correct	x		Contour	piano tones	9	Albouy *et al.* 2013^22^
					4ST	54.91	93.27	% correct	x					Albouy *et al.* 2013^22^
					5ST	64.7	95.47	% correct	x					Albouy *et al.* 2013^22^
300	300	0	2	3	2 ST	>70	>95	% H-F	x		Contour	pure tones	10	Gosselin *et al.* 2009^18^
				5		>40	>80	% H-F	x			pure tones		Gosselin *et al.* 2009^18^
540	500	40	3	5	1.8 ST	19,00	79,00	% H-F	x		Contour	piano tones	10	Tillmann *et al.* 2009^16^
540	500	40	3	5	1.8 ST	27.84	84.09	% H-F	x		Contour	piano tones	11	Albouy *et al.* 2013^23^

%H-F, percentage of Hits – False alarms; amusics are considered impaired compared to controls when the difference between the groups was significant.
