# Supplementary material for: Impaired encoding of rapid pitch information underlies perception and memory deficits in congenital amusia
Source: Sci Rep. 2016 Jan 6;6:18861. doi: 10.1038/srep18861 (PMC4702148; doi:10.1038/srep18861)
Supplement: Supplementary Information [file srep18861-s1.pdf]

Supplementary information

Impaired encoding of rapid pitch information underlies perception and memory deficits in congenital amusia

Philippe Albouy [1, 2, 3, 4], Marion Cousineau [3], Anne Caclin [1,2], Barbara Tillmann [1,2*] and Isabelle Peretz [3*]

[*]Both authors contributed equally to this work.

Affiliations:

[1] Lyon Neuroscience Research Center, Auditory Cognition and Psychoacoustics Team & Brain Dynamics and Cognition Team, CRNL, CNRS UMR5292, INSERM U1028, Lyon, F-69000, France

[2] Université Lyon 1, Lyon, F-69000, France

[3] International Laboratory for Brain, Music and Sound Research (BRAMS), Université de Montréal, Montreal, QC, Canada H3C 3J7

[4] Montreal Neurological Institute, McGill University, Montreal, QC Canada H3A 2B4

Corresponding author: Philippe Albouy, Montreal Neurological Institute, McGill University, 3801 University Street, Montreal, QC H3A 2B4 Canada. E-mail: phv.albouy@gmail.com Phone: +1 514.398.3973

Figure 1

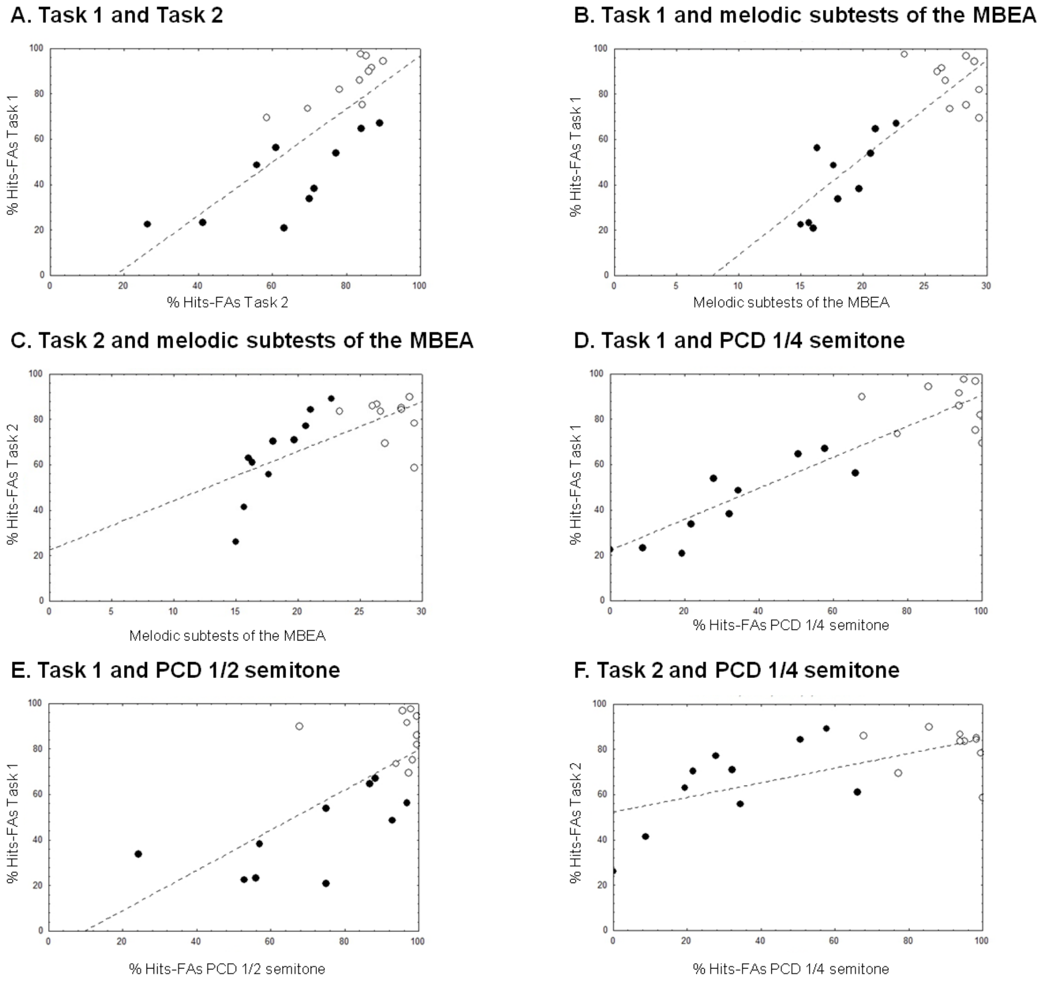

Figure 1: Scatter plot of amusic (black circles) and control (white circles) participants' performance: A. Performance in Task 1 (%Hits-FAs: average of all conditions) against performance in Task 2 (%Hits-FAs: average of all conditions); B. Performance in Task 1 (%Hits-FAs) against performance in the melodic subtests of the MBEA (average of the scale, interval and contour tests; maximum score = 30). C. Performance in Task 2 against performance in the melodic subtests of the MBEA. D. Performance in Task 1 against performance in the PCD task for the 1/4 semitone interval (%Hits-FAs, see Appendix Table 1). E. Performance in Task 1 against performance in the PCD task for the 1/2 semitone interval. F. Performance in Task 2 against performance in the PCD task for the 1/4 semitone interval. All correlations are significant in all participants and in amusic participants. Note that the correlation between performance in Task 1 and 2 is significant in all participants, and in amusics and controls separately.

Table A

Table A. Individual data for amusics (A) and controls (C) on the pre-tests and Task 1. Montreal Battery of Evaluation of Amusia (MBEA) Global[1] and Melodic[2] data are mean scores out of a maximum of 30, while Pitch Change Detection (PCD) and Task 1 accuracy data are % Hits - % False Alarms[3] as a function of pitch interval size in semitones (ST), and for Task 1, tone duration in ms.

	Pre-tests							Task 1			
	MBEA		PCD					1ST		2ST	
	Global	Melodic	1/4ST	1/2ST	1ST	2ST	3ST	100 ms	350 ms	100 ms	350 ms
A1	22.2	22.7	57.8	88.3	93.9	93.9	88.3	21.9	81.3	68.8	96.9
A2	15.3	15.0	0.0	52.8	100.0	97.2	100.0	12.5	34.4	-9.4	53.1
A3	21.8	19.7	32.1	57.1	82.1	93.2	96.0	6.3	56.3	12.5	78.1
A4	17.0	16.3	66.1	96.7	99.4	99.4	99.4	0.0	93.8	34.4	96.9
A5	19.0	17.7	34.4	92.8	98.3	98.3	98.3	18.8	68.8	18.8	87.5
A6	18.2	16.0	19.4	75.0	97.2	100.0	100.0	-15.6	50.0	-6.3	56.3
A7	23.8	21.0	50.6	86.7	86.7	92.2	92.2	37.5	71.9	50.0	100.0
A8	20.8	20.7	27.8	75.0	94.4	97.2	100.0	31.3	75.0	21.9	87.5
A9	20.0	18.0	21.7	24.4	52.2	88.3	88.3	-3.1	34.4	21.9	81.3
A10	18.8	15.7	8.9	56.1	89.4	95.0	95.0	-18.8	53.1	-9.4	68.8
C1	27.0	28.3	98.3	95.6	98.3	98.3	98.3	93.8	100.0	93.8	100.0
C2	26.0	27.0	77.2	93.9	99.4	99.4	99.4	50.0	84.4	59.4	100.0
C3	27.3	29.0	85.6	99.4	99.4	99.4	99.4	84.4	100.0	96.9	96.9
C4	25.5	26.3	93.9	96.7	93.9	96.7	96.7	78.1	90.6	96.9	100.0
C5	28.0	29.3	99.4	99.4	99.4	99.4	99.4	65.6	87.5	78.1	96.9
C6	28.0	29.3	100.0	97.2	100.0	100.0	100.0	18.8	75.0	90.6	93.8
C7	24.8	23.3	95.0	97.8	97.8	97.8	97.8	93.8	100.0	96.9	100.0
C8	26.2	26.0	67.8	67.8	67.8	65.0	67.8	78.1	100.0	81.3	100.0
C9	27.2	26.7	93.9	99.4	99.4	99.4	96.7	68.8	100.0	75.0	100.0
C10	27.5	28.3	98.3	98.3	98.3	98.3	98.3	43.8	96.9	68.8	90.6

[1]Global = mean score across the six sub-tests of the MBEA

[2]Melodic = mean score on the three melodic sub-tests of the MBEA (Scale, Contour, and Interval)

[3]Hits = correct responses on different trials, False Alarms = incorrect responses on same trials

Table B

Table B. Individual data for amusics (A) and controls (C) on Task 2. % Hits-FAs[1], accuracy presented as a function of sequence length (3, 4 tones), tone duration (100, 350 ms), Inter Tone Interval (ITI, 0, 250, 350 ms) and SOA (d+ITI).

	Task 2: % Hits-FAs for 3-tone sequences				Task 2: % Hits-FAs for 4-tone sequences			
d in ms	100		350		100		350	
ITI in ms	0	250	0	350	0	250	0	350
SOA in ms	100	350	350	700	100	350	350	700
A1	93.3	100.0	100.0	100.0	50.0	87.5	87.5	93.8
A2	50.6	25.0	49.0	3.9	31.3	12.5	37.5	0.0
A3	53.3	93.8	80.0	86.7	31.3	68.8	62.5	93.8
A4	40.0	75.0	73.3	93.3	18.8	81.3	37.5	68.8
A5	25.5	37.5	75.7	76.5	37.5	62.5	81.3	50.0
A6	40.8	68.8	53.3	86.7	25.0	62.5	68.8	100.0
A7	60.8	93.8	100.0	100.0	43.8	75.0	100.0	100.0
A8	66.7	93.8	80.0	82.4	37.5	56.3	100.0	100.0
A9	80.0	81.3	93.3	87.5	25.0	68.8	75.0	50.0
A10	48.2	50.0	40.0	60.8	6.3	25.0	43.8	56.3
C1	80.8	100.0	80.0	87.5	59.0	93.8	93.8	87.5
C2	40.0	93.8	66.7	93.3	31.3	75.0	68.8	87.5
C3	94.1	93.8	100.0	94.1	87.5	93.8	93.8	62.5
C4	68.2	100.0	100.0	100.0	56.3	93.8	93.8	81.3
C5	86.7	93.8	100.0	76.5	68.8	62.5	81.3	56.3
C6	53.3	75.0	60.0	73.3	12.5	56.3	68.8	68.8
C7	81.6	93.8	94.1	81.6	68.8	81.3	87.5	81.3
C8	87.5	87.5	94.1	94.1	75.0	75.0	87.5	87.5
C9	93.3	93.8	93.3	93.3	56.3	75.0	81.3	81.3
C10	80.0	93.8	100.0	100.0	62.5	81.3	75.0	81.3

[1]Hits = correct responses on different trials, False Alarms = incorrect responses on same trials
